# Laboratory-based X-ray phase-imaging scanner using Talbot-Lau interferometer for non-destructive testing

**DOI:** 10.1038/s41598-017-07032-y

**Published:** 2017-07-27

**Authors:** Shivaji Bachche, Masahiro Nonoguchi, Koichi Kato, Masashi Kageyama, Takafumi Koike, Masaru Kuribayashi, Atsushi Momose

**Affiliations:** 1 Institute of Multidisciplinary Research for Advanced Materials, Tohoku University, 2-1-1 Katahira, Aoba-ku, Sendai, Miyagi 980-8577 Japan; 2 Rigaku Corporation, 3-9-12 Matsubara-cho, Akishima-shi, Tokyo, 196-8666 Japan

## Abstract

An X-ray Talbot-Lau interferometer scanning setup consisting of three transmission gratings, a laboratory-based X-ray source that emits X-rays vertically, and an image detector on the top has been developed for the application of X-ray phase imaging to moving objects that cannot be tested clearly with conventional absorption contrast. The grating-based X-ray phase imaging method usually employs a phase-stepping (or fringe-scanning) technique by displacing one of the gratings step-by-step while the object stays still. Since this approach is not compatible with a scanner-type application for moving objects, we have developed a new algorithm for achieving the function of phase-stepping without grating displacement. By analyzing the movie of the moiré pattern as the object moves across the field of view, we obtain the absorption, differential phase, and visibility images. The feasibility of the X-ray phase imaging scanner has been successfully demonstrated for a long sample moving at 5 mm/s. This achievement is a breakthrough for the practical industrial application of X-ray phase imaging for screening objects carried on belt-conveyers such as those in factories.

## Introduction

X-ray phase imaging has been attracting attention because it produces significant contrast for weakly absorbing objects, such as polymers and soft biological tissues^1–4^. Among the methods of X-ray phase imaging reported so far, X-ray grating interferometry, such as X-ray Talbot interferometry^5^ and X-ray Talbot-Lau interferometry^6^, which is an advanced form of X-ray Talbot interferometry, is especially effective for practical X-ray phase imaging in clinical and industrial fields because they operate with conventional X-ray generators in hospitals and factories.

A Talbot or Talbot-Lau interferometer detects the slight X-ray refraction caused by an object. When the object is placed in the interferometer, the intensity change is detected through the gratings at each pixel of an image detector. This feature can be seen as a moiré pattern. X-ray phase imaging with interferometers is normally performed by analyzing moiré patterns acquired through a specific technique; that is, a fringe-scanning or phase-stepping technique by displacing one of the gratings. Moiré fringes contain the information of attenuation, phase shift (refraction), and scattering by an object; they appear as the changes of fringe intensity, fringe position, and fringe visibility^7^, respectively. X-ray phase imaging separates these information quantitatively and generates absorption, refraction, and scattering (in other words, visibility) images.

In the fringe-scanning procedure in combination with the grating displacement, the object is assumed to stay still in the field of view (FOV) during the entire scan, which is typically 30 s in the clinical application of X-ray phase imaging reported in ref. 8. However, in non-destructive testing for industrial production, it is desirable to scan products that move continuously on a belt-conveyor system. Fringe scanning by grating displacement is not compatible with such an industrial application.

This paper proposes an approach of a fringe-scanning method without any grating displacement for moving objects in the configuration of an X-ray Talbot-Lau interferometer. An apparatus (X-ray phase scanner) consisting of an X-ray source that emits X-rays vertically, X-ray transmission gratings, a sample stage that moves objects horizontally like a belt-conveyer, and a photon-counting image detector have been developed to demonstrate the approach. A pilot experiment of successful X-ray phase imaging for moving objects larger than the FOV of the apparatus is presented.

## Apparatus and Method

### X-ray phase scanner system configuration

**Figure 1:**
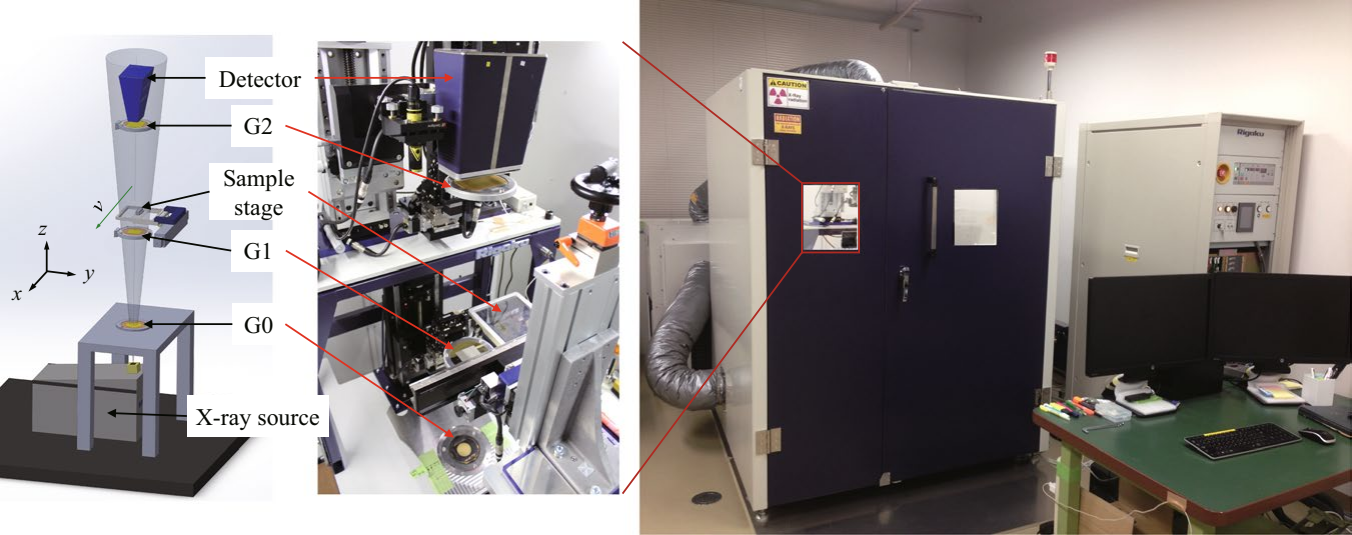
Schematic of the X-ray phase scanner prototype system (left) and its pictures (right).

An X-ray generator consisting of a tungsten rotating anode (UltraX 18, Rigaku, Japan) was placed at the bottom of the scanner to generate cone-beam X-rays in the vertical direction. The generator can be operated at up to 60 kV tube voltage and 90 mA tube current (5.4 kW maximum load). The focus size on the anode surface was 0.3 mm × 3 mm and the effective X-ray source size was 0.3 mm × 0.3 mm, taking into account the take-off angle (6°). The X-ray source size does not satisfy the requirement of spatial coherence for a Talbot interferometer, and therefore Talbot-Lau configuration with a source grating (G0), π/2-type phase grating (G1) and absorption grating (G2) (Microworks GmbH, Germany) was employed. The gratings were mounted and aligned using mechanical stages, as shown in Fig. 1. The periods of the gratings were 8.09 µm, 4.12 µm, and 8.4 µm, respectively, and assuming the design X-ray energy for the interferometer to be 30 keV, the G0-G1 and G1-G2 distances were 397 mm and 417 mm, respectively, which is reasonably compact for a scanner. The heights of gold pattern for G0 and G2 were 115 µm and 136 µm, respectively. The G1 was made of Ni and its height was 5.21 µm. The sample stage was located between G1 and G2 (68 mm above G1) and the sample was moved horizontally across the FOV. The gratings had line-and-space patterns and the grating lines were aligned parallel to the sample movement direction.

An image detector (Hypix-3000, Rigaku, Japan) was mounted on the top of the scanner to capture a movie of the moiré pattern while the sample is moved across the FOV. The detector is a photon-counting two-dimensional hybrid pixel array semiconductor detector having an active area of 77.5 mm × 38.5 mm with a pixel size of 100 μm × 100 μm. The detector has an X-ray photon count rate of greater than 10^6^ cps/pixel with a readout speed up to 174 fps frame rate.

### Phase measurement algorithm

An approach for the fringe-scanning method without grating movement has been reported by generating artificial moiré fringes with the assumption that the gratings are ideally fabricated and the spacing of the moiré fringes is constant^9–11^. However, the actual gratings used for a Talbot-Lau interferometer have slight deformations due to the fabrication process and mounting, and the degradation in resultant image quality is not negligible. It is not easy to fabricate an ideal grating, especially when a large area is required. In this study, we propose an algorithm that is applicable even when the gratings are deformed. Thus, the degradation of image quality is avoided, which leads to reduction in the cost of the apparatus.

The purpose of the algorithm is to calculate three resultant images, namely, absorption *A*(*p, q*), refraction *φ*(*p, q*), and visibility *V*(*p, q*) from a recorded moiré movie of sample scanning. Here, *p* and *q* are the image coordinates fixed to the moving sample in the *x-* and *y*-directions, respectively, as shown in Fig. 1. Note that we define (*p, q*) on the detector plane, and therefore, the coordinates on the sample are calculated by considering the magnification due to a cone-beam projection. The movement of a sample is assumed to be parallel with the *x*-axis in this setup. Therefore, *x* = *p*−*vt* and *y* = *q*, where *v* is the nominal sample speed on the detector plane and *t* is time.

**Figure 2:**
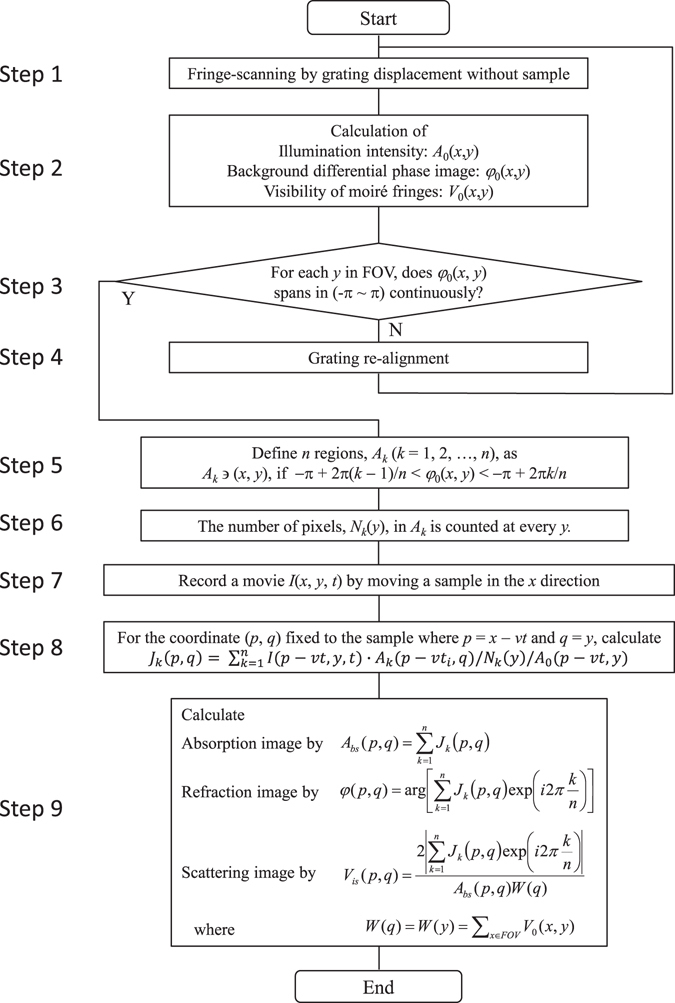
Flowchart illustrating the proposed algorithm for calculating absorption, refraction, and visibility images of a sample moving across FOV.

As a pre-processing, the effect of the built-in moiré pattern, which is normally observed without a sample because of incomplete grating alignment and slight grating deformation, was measured in advance as an instrumental function. For this purpose, we employed the conventional fringe-scanning technique or modified robust fringe-scanning technique explained elsewhere^12^ with grating displacement without a sample. Given the moiré pattern observed at the *j*-th step of a fringe-scan is *I*_0*j*_(*x*, *y*),

1 $${A}_{0}(x,y)=\sum _{j=1}^{M}{I}_{0j}(x,y),$$

2 $${\phi }_{0}(x,y)=\frac{{d}_{1}}{2\pi {z}_{0}}\,\text{arg}\,[{S}_{0}(x,y)],$$

3 $${V}_{0}(x,y)=\frac{2|{S}_{0}(x,y)|}{{\sum }_{j=1}^{M}{I}_{0j}(x,y)},$$

where

4 $${S}_{0}(x,y)\equiv \sum _{j=1}^{M}{I}_{0j}(x,y)\exp (-\frac{2\pi ij}{M}),$$

are calculated, as shown in Fig. 2 (Steps 1 and 2). *d*_1_ is the period of G1, *z*_0_ is the distance between G1 and G2 and *M* is the number of phase steps (*M* ≥ 3). *A*_0_(*x*, *y*) is illumination intensity, *φ*_0_(*x*, *y*) is the background differential phase caused by the deformation and alignment of gratings, and *V*_0_(*x*, *y*) is the visibility of moiré fringes. Note that *φ*_0_(*x*, *y*) is wrapped between −π to π, and phase unwrapping is not needed here.

As explained later, this algorithm assumes that every coordinate point (*p*, *q*) of the sample must travel across at least one moiré fringe along the *x*-axis. Therefore, *φ*_0_(*x*, *y*) must have values spanning from −π to π at every *y* (Step 3). If this condition is not satisfied, gratings are re-aligned, for examply by inclining one of the gratings, so that more moiré fringes are introduced, and steps 1 and 2 are repeated. After *φ*_0_(*x*, *y*) is thus defined, the FOV is divided into *n* regions, so that the pixel (*x*, *y*) having the value of *φ*_0_(*x*, *y*) between −π + 2π(*k* − 1)/*n* and −π + 2π*k*/*n* belongs to the *n*-th region. Further, flag matrices *A*_*k*_ (*k = *1, 2, *…, n*) (Step 5) are defined as,

5 $${A}_{k}(x,y)=\{\begin{array}{cc}1 & if-\pi +\frac{2\pi (k-1)}{n} < {\phi }_{0}(x,y)\le -\pi +\frac{2\pi k}{n}\\ 0 & otherwise\end{array}.$$

The number of flagged pixels in *A*_*k*_ at every *y* is stored in *N*_*k*_(*y*) (Step 6). The number *n* must be more than three and should be selected as large as possible, provided that sufficiently large values for *N*_*k*_(*y*) are available.

After this preparation, a movie is recorded while the sample moves linearly in the *x*-direction across the FOV (Step 7). Giving *I*(*x, y, t*_*i*_) as the movie frame at *t*_*i*_, where *i* indicates the frame number (*i* = 1, 2, … *m*), *J*_*k*_ (*p*, *q*) is calculated (Step 8) by

6 $${J}_{k}(p,q)=\sum _{p-v{t}_{i}\in FOV}I(p-v{t}_{i},q,{t}_{i})\cdot {A}_{k}(p-v{t}_{i},q)/{N}_{k}(q)/{A}_{0}(p,q).$$

**Figure 3:**
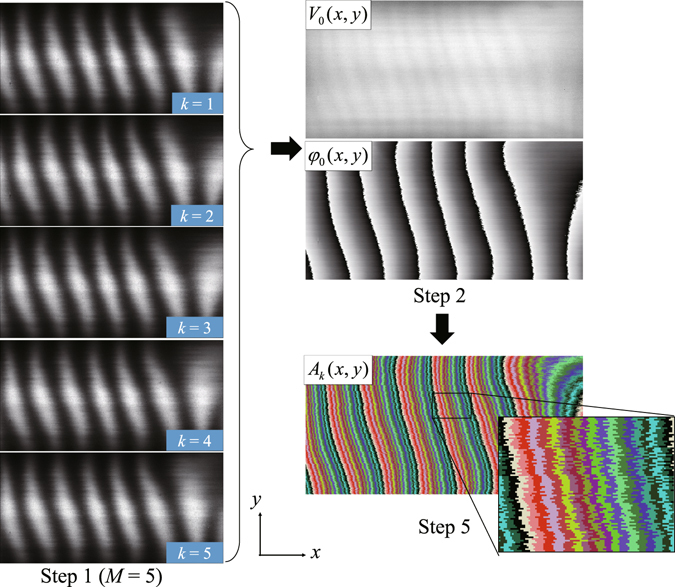
Graphical illustration from Step 1 to Step 5 shown in Fig. 2. *V*_0_(*x*, *y*) and *φ*_0_(*x, y*) are measured from moiré images (Step 1 to Step 2). The image labeled by *A*_*k*_ shows all regions (*k* = 1, 2, …, *n*) together with different colors to which the flagged pixels in *A*_*k*_ belong. *n* = 20.

As mentioned above, the coordinate (*p*, *q*) is fixed to the sample and relates to the coordinate (*x*, *y*) in the FOV with *x* = *p−vt*_*i*_ and *y* = *q*. Thus, *J*_*k*_ (*p*, *q*), which corresponds to the *k*-th step of fringe-scanning, is obtained without moving gratings but rather by utilizing the movement of the sample. Next, the resultant images are calculated with conventional formulae (Step 9):

7 $$A\,(p,q)=\sum _{k=1}^{n}{J}_{k}(p,q),$$

8 $$\phi (p,q)=\text{arg}[\sum _{k=1}^{n}{J}_{k}(p,q)\,e{\rm{xp}}(-i2\pi \frac{k}{n})],$$

9 $$V(p,q)=\,\frac{2{\sum }_{k=1}^{n}{J}_{k}(p,q)\,\exp (-i2\pi \frac{k}{n})}{A(p,q)\cdot W(q)},$$

where,

10 $$W(q)=W(y)=\sum _{x\in FOV}{V}_{0}(x,\,y)$$

is introduced to compensate the non-uniform visibility, especially in the *y* (*q*) direction due to cone-beam illumination. The raw images with moiré fringes generated by the experimental setup and an example of the definition of *A*_*k*_ are shown in Fig. 3.

## Result and Discussion

In this demonstration, the X-ray generator was operated at 60-kV tube voltage and 70-mA tube current. Samples were moved at a speed of 5 mm/s continuously and movies were recorded at 14-ms X-ray exposure per frame (71 fps). These parameters were chosen so that the nominal speed of the sample at the detector plane was one pixel per frame. The mean visibility of moiré fringes (*V*_0_ given by Equation (3) and shown in Fig. 3) was 52% around the center of the FOV.

**Figure 4:**
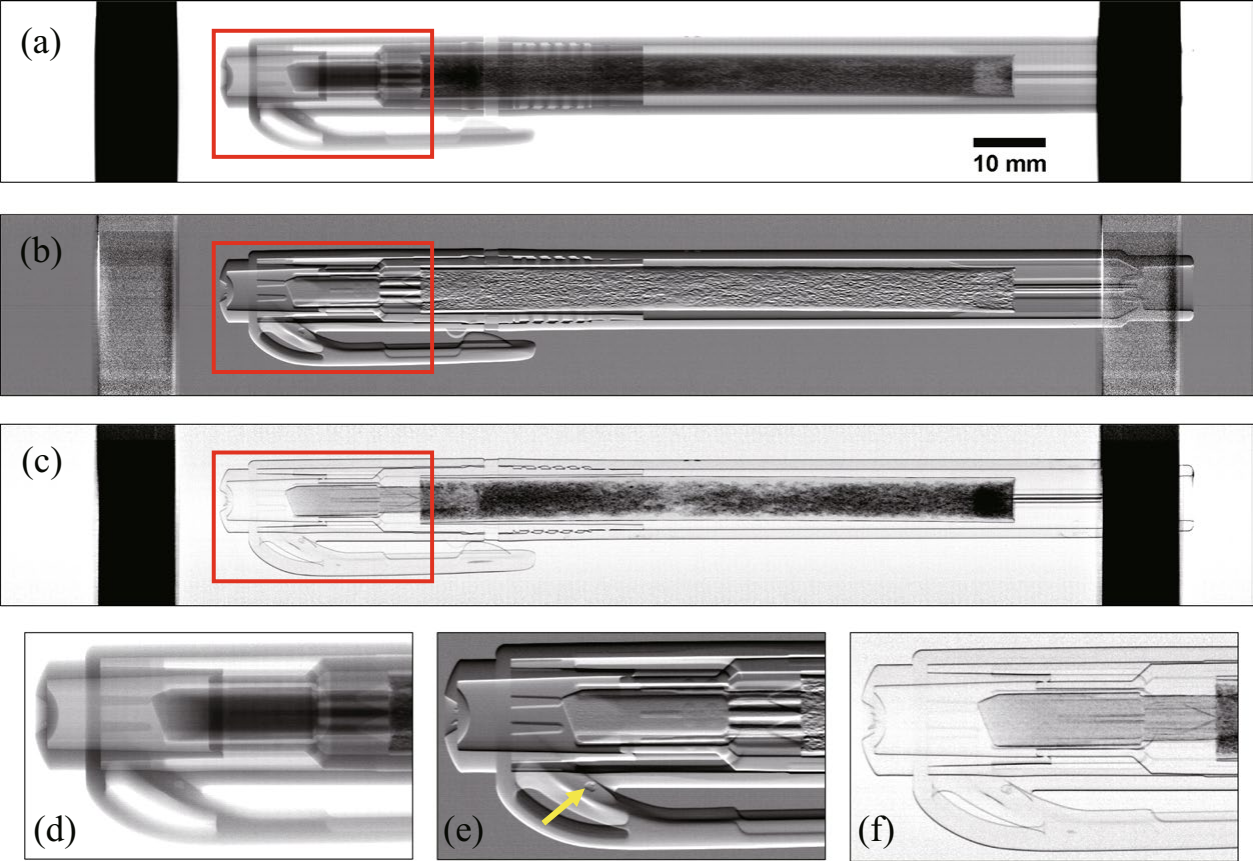
Results obtained for a highlighter pen by the proposed algorithms: (**a**) absorption image, (**b**) refraction image, and (**c**) visibility image. (**d**–**f**) are close-up images of the red rectangular areas shown in (**a**–**c**), respectively. The gray scales are set to (0.30, 0.42) transmittance for (**a**), (−2.08 µrad, 2.08 µrad) beam deflection for (**b**), and (0%, 50%) visibility reduction for (**c**). The arrow in (**e**) indicates an air bubble detected inside the cap.

Figure 4(a–c) show an absorption image, a refraction image, and a visibility image reconstructed for a highlighter pen. Figure 4(d–f) show close-up images of the red rectangular areas marked in Fig. 4(a–c), respectively. The lining structure, tip stoppers, cap locking mechanism, ink flow connector to the tip of the highlighter pen, sponge filled with ink and other small details are revealed in the resultant images. The contrast of the sponge filled with ink is especially remarkable in the visibility image as the contrast is considered to be generated by the ultra-small-angle scattering depending on the amount of ink in the sponge. The refraction image provided a significant signal of an air bubble inside the plastic cap as shown in Fig. 4(e). The two vertical dark areas in these images are the shadows caused by the metallic components of the sample holder stage.

**Figure 5:**
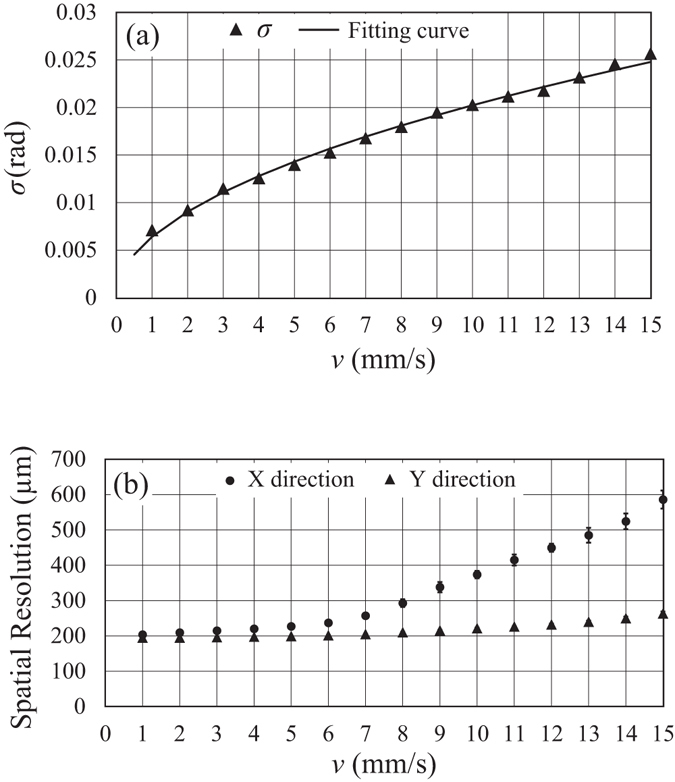
(**a**) The standard deviation *σ* of the refraction image, and (**b**) the spatial resolutions in *x-* and *y-*directions as functions of sample speed *v*.

The standard deviation *σ* of the refraction image calculated from 50 × 50 pixels near and outside of the sample in Fig. 4(b) was 0.014 rad in terms of moiré-fringe phase, which corresponds to 0.053-µrad beam deflection at the sample. Assuming that the *σ* is mainly caused by photon statistics, thanks to the low-noise property of the employed photon counting detector, the σ is theoretically proportional to the square root of total X-ray exposure, which is inversely proportional to the square root of sample speed *v* under a given X-ray intensity. Figure 5(a) shows the *σ* as a function of *v* and also a fitting curve by √*v* suggesting the validity of this assumption.

The sample speed is crucial to the spatial resolution in terms of motion blur. The spatial resolution at the detector plane in the *x*-direction was evaluated by the full width at half maximum (FWHM) of the differential edge profile of the sample feature in the absorption image. The spatial resolution in the *y*-direction, which was independent of the motion blur, was evaluated by the FWHM of a sharp edge profile in the refraction image. They are shown as functions of *v* in Fig. 5(b). As mentioned above, the sample speed of 5 mm/s corresponds to 1 pixel/frame; therefore, the effect of motion blur is not a concern below this speed in the *x*-direction. Actually, the spatial resolution near the Nyquist limit was attained below 5 mm/s, and clear degradation of the spatial resolution is found over that speed. Although the spatial resolution in the *y*-direction was almost constant as expected, the slight degradation shown in Fig. 5(b) is considered to be due to the effect of the vibration of the sample stage which increases with the sample speed. This was confirmed by evaluating the moiré movie of the sample scan where an increase in the sample vibration (below one pixel on average) was found with sample speed increment.

The apparatus presented here was developed mainly to demonstrate the function of the proposed algorithm to realize phase imaging for moving objects. In order to translate this result into the development of practical apparatuses for use in factories, various improvements are needed, such as increasing the sample speed and expanding the FOV. The spatial resolution due to vibration at higher speed could be improved, for example, by equipping a rigid and X-ray transparent stage, to which belt-conveyers are adjacent and products moving on the belt-conveyers are transferred smoothly. In order to increase the sample speed, detector sensitivity is essentially important. The detector employed in this study (Hypix-3000) was dedicated originally for low-energy X-ray experiments, which can be replaced for example with a CdTe-based photon counting image detector suitable for high-energy X-rays used for non-destructive testing. Fabrication of gratings with a high-aspect-ratio is also needed to enable the use of higher energy X-rays. While the height of the gold pattern of G2 was about 100 µm, a fabrication technique for realizing a height of 160 µm has been utilized for an experiment with 183-keV X-rays^13^. Thus, it is feasible to improve the apparatus design for a high-energy phase-imaging scanner.

In order to expand the FOV especially in the *y*-direction, larger gratings and a larger image detector are necessary. Fabrication of a large grating is a challenging task, but gratings of a 10 cm × 10 cm area^14^ have been fabricated. However, when a larger grating is employed with a cone-beam illumination, a curved form is required for a high-aspect-ratio grating, which creates another technical challenge. Instead, it is also possible to align multiple flat gratings and detectors to cover an area larger than the FOV of each grating-detector set.

Although the scanner was developed for imaging continuously moving objects, the concept can be applied for the relative movement between objects and the Talbot-Lau interferometer system. Therefore, scanning the Talbot-Lau interferometer with an X-ray source and detector against a stable object is feasible with the same concept of image formation. As a result, this study can be expanded to the examination of big structures that cannot be moved easily and to the human body for medical diagnoses.

## Conclusions

An X-ray phase-sensitive scanner with the Talbot-Lau interferometer configuration and a laboratory-based X-ray source was demonstrated. While conventional phase imaging is performed for an object stable in the FOV with the fringe-scanning technique, a special algorithm was proposed and implemented in this study for an object moving across the FOV. It should be noted that this algorithm allows the use of slightly deformed gratings. X-ray phase imaging was successfully performed for a sample moving at 5 mm/s with a spatial resolution of about 200 µm, which corresponded to the Nyquist frequency of the detector. Thus, the presented X-ray scanning setup is promising for practical use of X-ray phase imaging at factories for quality control of products moving on a belt-conveyor system.
